# Reviewed article: Silva RES, Lima EA, Silva AVL, Sousa JF, Oliveira EAR, Silva DMC, Carvalho MF, Carvalho RBN. Individual and contextual characteristics associated with concurrent risk factors among adolescents: a multilevel cross-sectional study based on the 2019 Brazilian National Health Survey. Epidemiol Serv Saude. 2025:34;e20240453.

**DOI:** 10.1590/S2237-96222025v34e20240453.a

**Published:** 2025-09-08

**Authors:** Sidiany Mendes Pimentel

**Table:** 

Rating	Excellent	Good	Average	Below average	Poor
Interest	✓				
Quality	✓				
Originality		✓			
Overall		✓			

**Table:** 

Questionnaire	Yes	No	Not applicable
Does the manuscript contain new and significant information to justify publication?	✓		
Does the Abstract (Summary) clearly and accurately describe the content of the article?	✓		
Is the problem significant and concisely stated?	✓		
Are the methods described comprehensively?	✓		
Are the interpretations and conclusions justified by the results?	✓		
Is adequate reference made to other work in the field?	✓		

## Manuscript Structure

Length of article is: Adequate

Number of tables is: Adequate

Number of figures is: Adequate

Please state any conflict(s) of interest that you have in relation to the review of this paper (state “none” if this is not applicable): None

## Comments

A proposta do artigo é clara. A temática é atual, bem fundamentada e apresenta uma análise bem estruturada. A metodologia utilizada é detallhada.

Na discussão, o trecho “Convém destacar que os resultados deste estudo convergem com os achados da Pesquisa Nacional de Saúde Escolar (PENSE) 2015, que apontou uma relação direta entre consumo de ultraprocessados, nível socioeconômico e escolaridade materna (34), sugerindo que menores vulnerabilidades facilitam o acúmulo de fatores de risco para doenças crônicas não transmissíveis (35), com destaque para a crescente exposição, fácil acesso e consumo desses alimentos nos estratos mais vulneráveis, tendendo à estabilidade entre os mais favorecidos (35)” provoca uma compreensão ambigua, necessitando de nova redação. 

